# The Story of the Insane from Year to Year

**Published:** 1899-06-03

**Authors:** 


### THE STORY OF THE INSANE FROM YEAR TO YEAR.

(Eleventh Series.)

### GLAMORGAN COUNTY ASYLUM.

During the year 1897 there was a considerable increase in the number of patients in this asylum. On the first day of the year there were on the books the names of 1,379 patients, and by the last day of December the number had risen to 1,484. The first admissions reached the respectable figure of 375, the readmissions 42, the recoveries 118, and the deaths 141. Calculated on the year's admissions the recoveries stand at 29·6 per cent., which is just a trifle below the Glamorganshire average for the last thirty years, and the deaths, calculated on the average number resident, stand at 7·8 per cent. Both the recovery rate and the death rate are below the county asylum average. Not fewer than 60 of the deaths were caused by cerebral and spinal diseases (general paralysis alone being responsible for 48), consumption for 23, and pneumonia for 5. Of the cases admitted during the year 96 owed their insanity to various moral causes, including domestic trouble, mental anxiety, and religious excitement. Of the physical causes intemperance in drink heads the list with a total of 122, and hereditary influence treads close on its heels with 118. Or, to put it another way, 240 of the 375 patients admitted to the asylum would never have been there at all had men and women reasoned before they married, and had abstained from alcoholic drinks. Dr. Pringle thinks, and no doubt thinks correctly, that insanitary surroundings contribute to swell the ranks of the insane. He says, "When one sees, even in the homes of the wealthy, no provision either for letting fresh air in or impure air out, it need not be a matter for surprise that in the homes of the working classes the only ventilation is by doors and windows, which practically means that during the winter months the same air is breathed over and over again, a lower vitality results, and, too frequently, a craving for alcoholics, owing to the sense of temporary comfort and well-being that they give." The asylum is somewhat overcrowded, but a block is being erected, and also temporary accommodation for male and female patients; nevertheless, the room thus obtained will afford only temporary relief, and the visitors wisely recommend the erection of another asylum in the district of Merthyr or Pontypridd. Painful as it is to arrive at this conclusion, there cannot be a doubt that it is the only satisfactory one. The visitors say that Dr. Pringle, with his well-known skill and conspicuous administrative ability, watches over the interests of the asylums in the same efficient and satisfactory manner as he has done in years past. The weekly charge to the unions is 7s. 10½d. per head. There are a few private patients, who pay from 10s. 6d. to 28s. a week.

### THE EAST SUSSEX ASYLUM AT HAYWARD'S HEATH.

Here the numbers decreased from 875 to 845, but this decrease has, unfortunately, not been owing to any lack of admissions, but owing to the transference of patients under contract to Fisherton House Asylum and the City of Exeter Asylum. There were 200 first admissions, 63 readmissions, 62 recoveries, 25 discharged relieved, and 101 deaths. These figures show that the recoveries stand at 27·8 per cent. of the admissions, and the deaths at 11·4 per cent. on the average number resident. The recoveries are considerably below the county asylum average, and slightly below the Hayward's Heath Asylum, while the deaths are a little above it. Of the deaths, 56 were caused by cerebral and spinal diseases, 9 by pneumonia, 5 by phthisis, and 1 by enteritis. Various moral causes account for 29 of the year's admissions, intemperance in drink for 21, hereditary tendency for 57, previous attacks for 51, and 83 are returned as of unknown causes. Dr. Saunders seems to have had among his year's admissions a large proportion of troublesome and unsatisfactory cases, for we notice that 74 were actively suicidal on admission, and 56 were over 55 years of age. As regards 4 of these admissions it is noted that their combined ages amounted to no less than 302 years. There were 36 post-mortem examinations during the year. If this proportion could be relied on to include those in which such examinations are necessary, it would never need to be increased. This, of course, cannot always be the case, but it is better so than that the senseless boast of a post-mortem in every case be indulged in; and we are glad that Dr. Saunders says that "the personal feelings of the relatives are considered to be entitled to respect before even a mandate from a central authority, however good the intention may be for which this is issued." Six of the staff received pensions during the year; the staff had to be increased owing to increase in the number of patients, and there would be the usual number of resignations. All these, with the crowded state of the asylum, and the worry inseparable from drafting patients to other asylums, must have combined to make an anxious year for Dr. Saunders. Dr. Lockhart Robertson, the first superintendent, died during the year, shortly after having obtained his pension as Lord Chancellor's Visitor, and Dr. Saunders feelingly refers to the death in a well-expressed paragraph, concluding with the statement that Dr. Robertson placed the asylum, "by his scholarly attainments and reputation, in the forefront of similar institutions in this or any other country in the world." The weekly rate of maintenance is a trifle over 9s. 4d. per head.

### THE SUNDERLAND BOROUGH ASYLUM.

On January 1st, 1897, this institution contained 314 patients, and on the last day of December the number had fallen to 304. There were 93 first admissions, 13 readmissions, 29 recoveries, and 33 deaths. Calculated on the year's admissions, the recoveries stand at 29·5 per cent., and the deaths calculated on the average number resident at 10·8 per cent. Intemperance in drink is responsible for 23 of the admissions, and hereditary influence for 41. Of the deaths during the year, 17 were caused by cerebral and spinal diseases, and five by consumption. Unlike what is usually found in asylums, there were more men than women in residence at the close of the year, indeed, the male side was practically full, while the female side had 34 vacant beds. Although it is only in its fourth year of existence, it is clear that additions will soon have to be built. Training classes for the nurses are carried on by Dr. Yeates, the assistant medical officer, and nine of the staff presented themselves for examination for the certificate of the Medico-Psychological Association, and all obtained it. It is to be hoped that all these nine will continue their studies and obtain a higher nursing certificate. Of late years we have heard much of the advantages derivable from small wards in asylums, and unquestionably, as Dr. Elkins states, the patients can be better "individualised"; but as he also remarks, they are expensive and difficult to staff, as the nurses and attendants are very scattered, and we may add that charge nurses are incomparably more difficult to find than ordinary nurses. Probably wards containing 50 or 60 patients are the easiest to handle; and generally the best for the patients. During the year Dr. Elkins resigned his appointment at Sunderland on being appointed medical superintendent of one of the metropolitan asylums, and Dr. Middlemass was elected to the vacancy. We learn from the visitors' report that "Plans for the rearrangement of the drainage have been approved by the Secretary of State," and that the "system of heating and ventilation is also engaging the attention of the committee." This sounds strange when referring to a new asylum. The weekly rate is a fraction over 8s. 9d. per head.

